# Amitriptyline 2% cream vs. capsaicin 0.75% cream in the treatment of painful diabetic neuropathy (Double blind, randomized clinical trial of efficacy and safety)

**Published:** 2015

**Authors:** Javad Kiani, Saman Ahmad Nasrollahi, Farzaneh Esna-Ashari, Puyan Fallah, Firuzeh Sajedi

**Affiliations:** a *Division of Endocrinology, Department of Internal Medicine, School of Medicine, Hamedan University of Medical Sciences, Hamedan, Iran. *; b *Center for Research and Training in Skin Diseases and Leprosy (CRTSDL), Tehran University of Medical Sciences, Tehran, Iran.*; c *Department of Community Medicine, School of Medicine, Hamedan University of Medical Sciences, Hamedan, Iran.*; d *Department of Internal Medicine, School of Medicine, Hamedan University of Medical Sciences, Hamedan, Iran.*

**Keywords:** Capsaicin, amitriptyline, diabetic neuropathy, cream, pain

## Abstract

Because of less systemic side effects of topical medications in pain relief of the painful form of diabetic peripheral neuropathy, this study aimed to compare the effect of amitriptyline and capsaicin cream in relieving pain in this condition.

In this randomized, double-blind and non -inferiority trial, 102 patients received amitriptyline 2% and capsaicin 0.75% creams 3 times a day for 12 weeks on the feet. Pain relief was measured by the visual analog scale (0–10). Treatment responding was considered as cure rate greater than 50% from baseline. Evaluations of the pain severity, compliance and drugs adverse effects were performed at each of the 4-week follow -up visits.

Both drugs significantly relieved pain in 12 weeks compared with baseline values (*P *< 0.001 for both). Treatment responders were similar in both groups (*P *= 0.545). Intention-To-Treat analysis showed no significant difference in the efficacy between the two treatments (*P *= 0.703). Adverse events were more common in capsaicin group (P = 0.001). Dermatologic complications were the most common: itching, blister formation and erythema in the capsaicin group and skin dryness and itching in the amitriptyline group.

This study demonstrates the similar efficacy of amitriptyline cream with capsaicin cream in managing diabetic neuropathic pain with fewer side effects.

## Introduction

Diabetic peripheral neuropathy (DPN) is one of the most common, expensive, and disabling complications of diabetes (1). Previous studies have reported a worldwide prevalence of 22.7% to 54% for it (2). Painful form of this complication is very uncomfortable for patients and despite recent improvements in its treatment, the pain is often inadequately controlled (3). A wide variety of oral medications have been shown to considerably reduce neuropathic pain compared with placebo in randomized controlled trials (4). Because of less systemic side effects, use of topical medications in this field is more acceptable. The final goal of development of topical compounds is the better compliance to medical treatment, by providing efficient pain relief with less central nervous system effects and minimal drug regimen burden (5). Efficacy of topical capsaicin formulations those act by the reduction of substance P content in skin have demonstrated in controlled clinical trials (6-8). Amitriptyline is a tricyclic antidepressant that acts centrally by inhibiting neuronal reuptake of norepinephrine and serotonin and is used effectively in many chronic neuropathic pain conditions (9-11). But its adverse effects such as sedation, postural hypotension, and anticholinergic effects, in oral administration have limited titration to higher doses needed to achieve adequate analgesia (12). Topical form of this drug is used in some studies for the treatment of neuropathic pain (12-14). Given the greater tendency to use of local medications in this area, this study aimed to compare the effect of amitriptyline and capsaicin cream in relieving pain associated with DPN.

## Experimental

*Study participants*

Patients with type 2 diabetes, aged between 30 and 70 years, who had DPN were considered for the study. Patients with chronic daily pain for more than three months, who had a pain score of at least 4 as assessed by visual analog scale (VAS), were enrolled in the study. Patients with less than one year duration of diabetes, opium or alcohol use, other causes of neuropathy, hepatic or renal failure (serum creatinine > 1.5 mg/dl), clinically significant cardiovascular disease, HA1C ≥ 9%, pregnancy or lactation, ulcer or infection of foot and hypersensitivity to pepper were excluded from the study. Use of any medication for neuropathic pain was discontinued two weeks before enrolling in the study and throughout it, but required therapies including insulin and oral hypoglycemic agents were maintained throughout the study.

*Study design and treatment*

This study was approved by Hamedan University of Medical Sciences Research Ethics Committee (D/P/16/35/9/1282) and had been registered in IRCT (Registration ID: IRCT201209238308N2). This 12-week, randomized, double-blind, parallel-group and non-inferiority trial was conducted to compare the efficacy and safety of amitriptyline and capsaicin for pain associated with DPN. Laboratory tests, physical examinations, body weight, height, blood pressure and general characteristics including age, duration of diabetes, history of hypertension, pain duration and medications were recorded at the base of the study. PDN was confirmed by NSS (Neuropathy Symptom Score) and NDS (Neuropathy Disability Score) criteria (15, 16). Patients were randomized to amitriptyline or capsaicin group. Randomization was done by permuted-block design that involves randomizing patients in treatment groups in sequential blocks. Patients were then treated under double-blind conditions for up to 3 months. Written informed consent was obtained from each patient prior to enrollment.

The study drugs were amitriptyline 2% cream and capsaicin 0.75% cream in no label laminated tubes containing 100 gram of material. In order to fabricate amitriptyline oil/water cream, both Lipid phase (including isopropyl myristate, Cetyl palmitate, Stearyl alcohol, Cetyl alcohol and Span 60) and aqueous phase (contained of Amitriptyline, Tween 80 and distilled water) were separately heated to 75ºC. After that, the hot water phase was added to the lipid phase under stirring (600 rpm) and cooled down to 25ºC. We used 2% concentration of the amitriptyline cream because a few controlled clinical trials examined previously this concentration and had demonstrated its efficacy and safety (13, 14, 17). Amitriptyline active ingredient was obtained from Iran Daru Pharmaceutical Company (Iran) and capsaicin was provided by Kish Medipharm pharmaceutical Company (Iran)**.** Physicochemical and microbial tests of amitriptyline cream were assessed by ICH methods and USP guidelines. Formulation was kept at 45ºC and 75% humidity for 6 months (accelerated conditions) and during this period, stability data were getting at month 1, 3 and 6. No changing in pH, viscosity, density, formulation color, odor and etc was seen. Also total bacterial count (CFU/g) and total fungi and yeast count (CFU/g) were determined. 

Amitriptyline or capsaicin was dispensed to patient according to randomized group assignment. Patients applied either of drugs topically below the ankles on the feet 3 times a day.

The end point of the study was the reduction in the median pain score from baseline, as assessed by the VAS (0-10 points). Evaluations of the patients including assessment of the pain severity, vital signs and examination and questioning regarding the adverse effects were performed at each of the 4-week follow-up visits. Compliance was assessed by direct questioning. The investigator was accessible by telephone to all patients throughout the study.

*Statistical analysis*

The efficacy analyses were performed on both per protocol analysis and the intention-to-treat (ITT) analysis. ITT population was defined as randomized patients who took at least one dose of medication and provided at least one baseline and one post-baseline efficacy assessment. The efficacy endpoint was the change in mean monthly pain score on the Visual Analogue Scale (VAS). Values are expressed as means ± SD and numbers and percentages. The patient VAS was compared using the Student t test. Cure rates between treatment groups and incidence of adverse events were compared by Chi-Square Test. Repeated measure analysis was performed on VAS change scores (from baseline to week 12). Baseline parameters were compared by the Student t test, Mann-Whitney or Chi-Square tests. Missing data for subjects who terminated early were imputed using multiple imputations by regression method. A P value < 0.05 was considered significant. SPSS (version 20.0) was used to perform analysis.

## Results and Discussion

The study was conducted between February 2013 and January 2014. Patients’ demographic and baseline characteristics were comparable between two groups of treatments as presented in Table 1. The flow chart of patient enrollment and disposition is shown in Figure 1.197 eligible 

patients of 354 screened patients were enrolled in the study. The ITT population for the efficacy analysis consists of 51 patients receiving amitriptyline and 51 patients receiving capsaicin. 64 patients of a total of 102 completed the study with 62.7% compliance. A total of 38 (37.2%) patients discontinued therapy during the treatment period: 16 (31.3%) taking amitriptyline and 22 (43.1%) taking capsaicin (*P *= 0.219).

**Table 1:** Demographic and baseline characteristics of patients in capsaicin and amitriptyline groups.

**Variable**	**Capsaicin group**	**amitriptyline group**	**P-value**
Age, years (SD)	55.4±10.6	57.5±10.8	0.314
Gender			
Female, n (%) Male, n (%)	35(68.6) 16(31.4)	34(66.7) 17(33.3)	0.832
Height (cm)	163.3±7.9	162.0±7.4	0.644
Weight (kg)	74.4±11.3	72.9±12.9	0.524
Body Mass Index (Kg/m2)	27.5±3.4	27.4±4.1	0.894
Duration of diabetes (years)	8.0±5.1	9.0±6.7	0.864
HbA1C	7.2±0.89	7.5±1.1	0.177
Creatinine	0.9±0.19	1.0±0.25	0.055
Medication (%) Oral agent Insulin Oral agent + Insulin	24(47.1) 16(31.4) 11(21.6)	28(54.9) 9(17.6) 14(27.5)	0.269
Pain Duration (month)	19.0±18.3	18.9±15.3	0.763
Mean basal VAS	7.4±1.4	7.8±1.7	0.172

**Figure 1:**
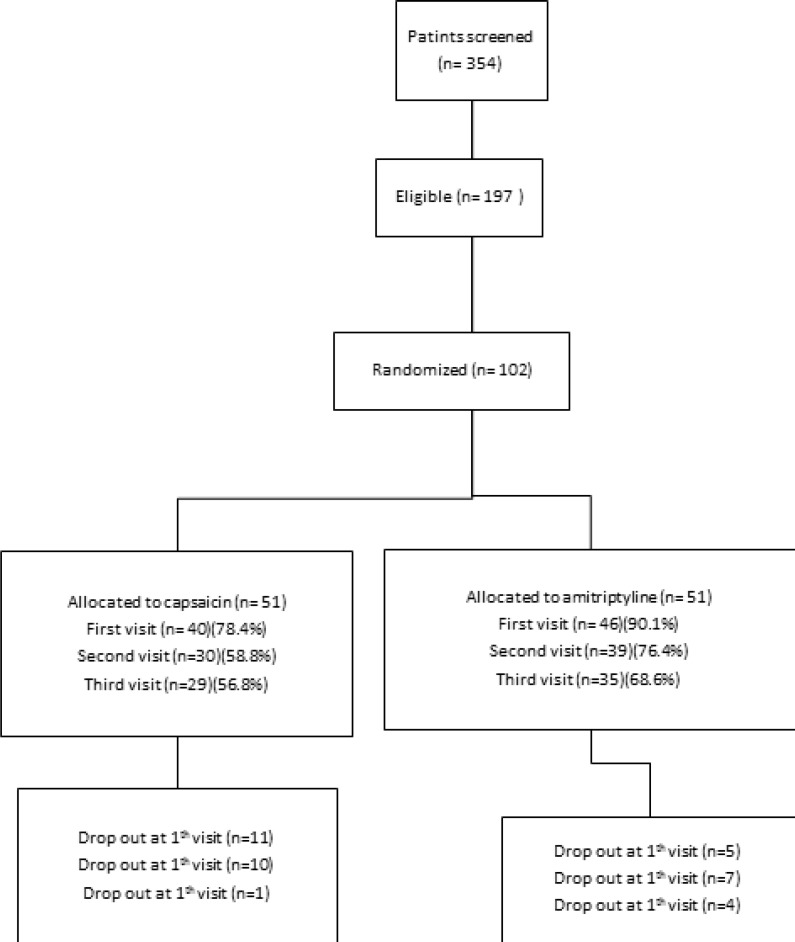
The flow chart of patient enrollment and disposition

Treatment responders (≥ 50% reduction from baseline on the VAS assessment) (18) in the ITT population, at week 12, were 22 (43.1%) and 19 (37.3%) of patients with amitriptyline and capsaicin, respectively (*P *= 0.545).

**Figure 2.**
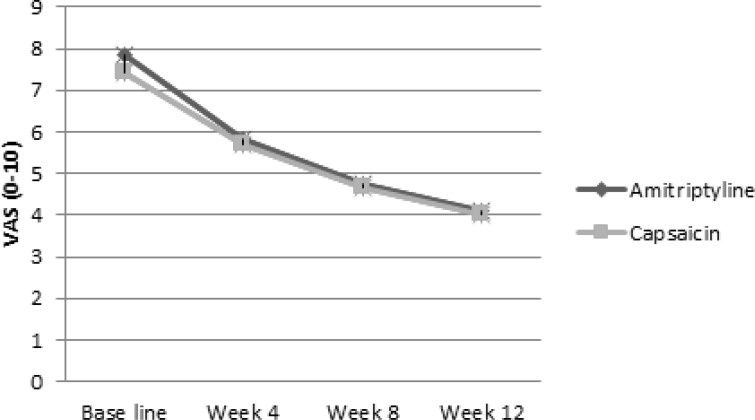
Efficacy of amitriptyline vs. capsaicin on pain severity over 12 weeks of treatment.

Pain in the intention to treat population decreased with time as shown in Figure 2 (p value < 0.001 for both of them). The slope of the pain decline in amitriptyline group (-1.26) was approximately similar to the capsaicin group (-1.13). There was no significant difference between the slopes of VAS decline between the two groups (Δ = 0.13, p = 0.703). Per protocol analysis showed similar results. In logistic regression analysis there was not any relationship between patient’s basic characteristics and response to treatment in amitriptyline and capsaicin groups.

**VAS: Visual Analogue Scale.**

Adverse events were more common in capsaicin group: 13 (25.5%) patients in amitriptyline group and 29 (56.9%) patients in capsaicin group (P = 0.001). Dermatologic complications were the most common reported adverse events. In the amitriptyline group skin dryness and then itching were the most common dermatologic adverse effects (8.8% and 4.4%, respectively). The most common local side effects of capsaicin were itching, blister formation and erythema respectively (20%, 8.5% and 5.7%).

This randomized, double-blind and non-inferiority, 12-week trial demonstrates the comparable efficacy of amitriptyline cream in comparison with capsaicin cream in the management of pain due to peripheral neuropathy in type 2 diabetic patients. In addition to relieving pain, treatment with amitriptyline cream was also associated with less adverse events.

A few studies evaluated the effect of topical amitriptyline in this field. A study by Kopsky described two cases of neuropathic pain of those treated effectively with topical amitriptyline 5% and 10% (19). In a randomized, double-blind and placebo controlled trial, the analgesic effects of 50 mmol/L and 100 mmol/L solutions of amitriptyline those evaluated in 14 healthy volunteers were significantly higher than those of the placebo (20). A cure rate of 43.1% and 25.5% adverse events for amitriptyline cream those were better than capsaicin shows the value of this drug for more research. Inconsistent with results of our study, an open label pilot study that examined the effect of topical amitriptyline, ketamine and a combination of both for neuropathic pain, didn’t show any significant difference from placebo for any treatment (21). Also, a double-blind, randomized, placebo-controlled crossover study that evaluated the efficacy of topical 5% amitriptyline and 5% lidocaine in treating of neuropathic pain failed to show efficacy of topical amitriptyline (12). In general, in our study amitriptyline was well tolerated and safe during this 12-week study. There were more discontinuations due to adverse events in the capsaicin treatment group than in the amitriptyline treatment group. This study compared the efficacy of amitriptyline cream with capsaicin cream, a FDA approved drug, but prolonged therapy and evaluation for a longer duration than 12 weeks can better evaluate the benefits of this drug. Strength of the current study includes the use of a randomized, prospective design, but measure of only one outcome (median pain score by VAS) is one of the limitations. Given the conflicting results of the previous studies, more studies are required to better evaluate the efficacy and safety of this topical compound for relieving pain in diabetic peripheral neuropathy.

## Conclusion

In sum, this study demonstrates that amitriptyline is effective in managing diabetic neuropathic pain similar to capsaicin cream with less side effects and better patient compliance. Treatment with topical amitriptyline was safe and without significant side effects associated with systemic therapies. Further studies are required to confirm the efficacy and safety of topical amitriptyline as a treatment of PDN.
